# Interferon regulatory factor 4 as a context-dependent regulator of inflammation: transcriptional and epigenetic control, skin diseases, and translational challenges

**DOI:** 10.3389/fimmu.2026.1954595

**Published:** 2026-09-21

**Authors:** Hui Zhang, Fang Cao, Jinghui Qian, E Liu, Ying Huang, Xueer Zhang, Kexin Tang, Jing Guo

**Affiliations:** 1 School of Clinical Medicine, Chengdu University of Traditional Chinese Medicine, Chengdu, China; 2 Dermatological Department, Hospital of Chengdu University of Traditional Chinese Medicine, Chengdu, China; 3 Endocrinological Department, Sichuan First Hospital of Traditional Chinese Medicine, Chengdu, China

**Keywords:** context-dependent transcriptional regulation, interferon regulatory factor 4 (IRF4), intracellular inflammatory mediator, skin disease, T helper 17 cells

## Abstract

Inflammatory mediators are usually defined as extracellular signals, yet intracellular regulators determine which signals are converted into cell-specific, durable inflammatory programmes. Interferon regulatory factor 4 (IRF4) is a stringent test of this broader concept because its effects vary with abundance, partner choice, chromatin accessibility, metabolism, cell lineage, tissue, and disease phase. We conducted a systematised narrative review and applied a causal-discrimination framework that separates lineage determination and activation from downstream amplification, necessary mediation, sufficiency, and therapeutic tractability. Across immune compartments, IRF4 supports pathogenic T helper 17 and type 2 programmes but also maintains regulatory T cells, humoral immunity, antitumour CD8 T-cell activity, intestinal group 3 innate lymphoid cells, and skin repair. Psoriasis provides the most developed disease-linked preclinical hypothesis, although the evidence is concentrated in one 2025 study and shows pre-induction benefit in an acute mouse model rather than reversal of established human disease. Atopic dermatitis remains a candidate epithelial and immune-network context without independent target validation. Conflicting evidence on IRF4 dependence in differentiated T helper 17 cells further shows that developmental requirement cannot be assumed to predict acute therapeutic sensitivity. We therefore define IRF4 as a context-encoded intracellular mediator only when reversible, context-resolved perturbation links target engagement to a prespecified molecular programme and disease-relevant phenotype. Human-tissue perturbation, cell- and complex-resolved target engagement, lesional pharmacokinetics and pharmacodynamics, reversibility, incremental value over established pathway control, and exposure-matched safety testing are the decisive gates for translation.

## 1 Introduction: expanding the concept of an inflammatory mediator

Inflammatory mediators are usually described as extracellular molecules that transmit signals between cells. This definition captures cytokines, chemokines, lipids, and other soluble factors, but it does not fully explain how an inflammatory response acquires cellular specificity, magnitude, persistence, or reversibility. These properties also depend on intracellular regulators that determine which mediator genes can be expressed in a given cell state. Interferon regulatory factor 4 (IRF4) is a strong test of this broader functional concept. IRF4 has relatively weak autonomous DNA-binding activity and acts mainly through cooperative transcriptional complexes whose composition differs amongst immune lineages (1–3). Its abundance varies with receptor-signal strength and influences the scale of lymphocyte expansion and differentiation (4–6). We therefore define an intracellular inflammatory mediator operationally: its perturbation must connect an upstream signal to a specified downstream inflammatory programme and a disease-relevant phenotype in the same context. Expression, network centrality, and developmental requirement alone do not satisfy this definition. We focus on IRF4 because it is a well-characterised signal-responsive transcription factor with distinct, experimentally defined functions across T-cell, dendritic-cell, myeloid and B-cell contexts; this breadth makes it a useful test case for distinguishing expression, lineage dependence and acute disease mediation. In this review, increased IRF4 expression is treated as an association only: it can reflect cell recruitment, activation or changes in cell composition and does not by itself show that IRF4 functionally drives inflammation.

IRF4 does not have one fixed inflammatory effect; its effect depends on the cell type, stimulus, tissue and disease stage. In activated T cells, IRF4 supports T helper 9 (Th9) and T helper 17 (Th17) differentiation, as well as cytotoxic T-cell expansion (2, 5, 7, 8). In macrophage systems, IRF4 has been linked both to restraint of Toll-like receptor signalling and to IL-4-conditioned transcriptional programmes (9–11). Other studies connect myeloid IRF4 to fibrotic injury, epithelial recovery, or wound repair, depending on the tissue and experimental phase (12–15). Dose-sensitive regulatory T-cell (Treg) homeostasis adds a further boundary to interpretations based solely on pathogenic effector cells (16). These findings need not represent a biological contradiction. They may reflect changes in lineage composition, activation state, binding partners, chromatin, tissue environment, or time. Some phenotypes may also arise because IRF4 is required for cell development, rather than because it acutely mediates an ongoing inflammatory response.

This review uses a practical evidence framework to distinguish six different claims: IRF4 may help establish a cell lineage, indicate cell activation, increase a downstream inflammatory response, be required for that response, be sufficient to start it, or be a realistic drug target. These are different claims and require different evidence. For example, high IRF4 expression or a developmental knockout can show association or lineage dependence, but neither alone proves that reducing IRF4 in an adult disease tissue will improve disease. A causal disease claim requires perturbing IRF4 in the same relevant context, observing the proposed downstream programme, and measuring a disease-relevant phenotype (16, 17, 55). Therapeutic targetability requires additional evidence of reversible adult modulation, target engagement in the intended compartment, dose-response, delivery, recovery after withdrawal, and exposure-matched safety assessment (15, 16, 28, 29, 37, 42, 55).

The synthesis positions IRF4 as a context-encoded transcriptional node rather than a predetermined pro-inflammatory or anti-inflammatory factor. Molecular partner choice, abundance, chromatin, metabolism, lineage, tissue, and disease phase specify the programme that IRF4 can execute. These variables explain why pathogenic dependencies coexist with homeostatic functions that may limit systemic inhibition. Within inflammatory skin disease, psoriasis represents the most developed disease-linked preclinical hypothesis identified in the accessible literature, based predominantly on one 2025 evidence package. It is not a clinically validated indication. Atopic dermatitis (AD) remains a less mature candidate network-amplifier context. We first define the molecular determinants of IRF4 activity, then examine lineage-specific circuits and liabilities, disease causality in skin, and the requirements for therapeutic tractability.

## 2 Methods

We conducted a systematised narrative review centred on PubMed/MEDLINE. Prespecified thematic query blocks covered IRF4 and inflammation, inflammatory skin disease, T-cell contexts, myeloid and dendritic-cell biology, transcriptional partners and chromatin, therapeutic approaches, and counterevidence. The original search was updated by targeted PubMed and publisher-record checks through 21 July 2026. Discovery was supplemented by backward and forward citation chaining. Foundational studies were retained when necessary to interpret recent mechanisms, whereas update searches prioritised work published from 2021 onward. Evidence was classified by source and directness, including human genetics, human tissue association, human ex vivo perturbation, mouse genetic perturbation, animal intervention, reductionist mechanism, and cross-tissue inference. Authenticated exportable searches of Embase, Web of Science, and Scopus were not available. Screening was purposive and relevance ranked, and complete prospective deduplication and exclusion counts were not recorded. This article is therefore a systematised narrative review, not a systematic review, and no PRISMA flow diagram is presented. The final narrative synthesis cites 61 publications: 46 addressing molecular or lineage biology (1–46) and 15 providing inflammatory skin-disease, therapeutic or comparative clinical context (47–61). These counts describe the final cited evidence base, not a systematic-review screening flow.

## 3 Molecular determinants of context-dependent IRF4 activity

### 3.1 Cooperative DNA recognition, abundance and partner choice

IRF4 contains an N-terminal DNA-binding domain and a C-terminal interferon-association domain connected by a regulatory region. Structural analysis identified a compact linker and a flexible autoinhibitory segment that constrain DNA binding and protein interactions (1). Unlike IRF3 and IRF7, IRF4 is not primarily organised to drive type I interferon induction. Its low intrinsic affinity for DNA makes cooperative recruitment central to target selection. In B-lineage cells, IRF4 can cooperate with ETS-family proteins at ETS–IRF composite elements. In activated T cells, BATF–JUN complexes recruit IRF4 to AP-1–IRF composite elements and enable a distinct transcriptional programme (2, 3). These binding modes explain how the same IRF4 protein can contribute to plasma-cell differentiation in one context and effector T-cell differentiation in another. Motif identity, however, is not a disease diagnosis. It identifies a possible regulatory complex whose activity must still be demonstrated in the relevant cell, tissue, and inflammatory state.

IRF4 abundance carries biological information in addition to marking activation. T-cell receptor stimulation induces graded IRF4 expression, and mouse studies have linked this gradient to the magnitude of CD8-positive T-cell expansion, metabolic adaptation, and effector differentiation (4–6). Higher and more sustained IRF4 abundance was associated with stronger effector programmes in these systems, whereas reduced IRF4 limited expansion or altered differentiation. The perturbational evidence supports an instructive role in the tested models, but two qualifications matter for translation. First, receptor strength and IRF4 abundance change together, so an observational increase cannot identify IRF4 as the independent cause of a human disease state. Second, complete genetic loss is not equivalent to partial suppression in an adult cell. A dose-encoded regulator may cross different functional thresholds in pathogenic and protective compartments. Preferential sensitivity of a pathogenic compartment nevertheless remains hypothetical until exposure-response relationships are measured with the same intervention.

Partner choice and post-translational state add further specificity. In murine polarised Th17 cells, a 2025 study linked FLI1–IRF4 cooperation to a subset of the effector transcriptional programme (17). Earlier work showed that ROCK2-dependent phosphorylation of IRF4 contributed to IL-17 and IL-21 production in autoimmune model systems (18). These findings support the possibility that an IRF4 programme might be modified at the level of a partner complex or molecular state rather than by eliminating the protein. They do not establish that such selectivity is pharmacologically attainable. A molecule that weakens BATF–JUN–IRF4 assembly, for example, would need direct occupancy and functional data before it could be assumed to spare ETS-associated B-cell programmes. Compensatory transcription factors, shared interfaces, and changes in chromatin accessibility could all reduce the expected selectivity.

### 3.2 Chromatin, metabolism and temporal state

IRF4 activity is also conditioned by chromatin and metabolism. During IL-4-induced macrophage activation, IRF4 expression participates in a wider programme that includes JMJD3-dependent removal of repressive H3K27 methylation and mTORC2-dependent metabolic reprogramming (10, 11, 19). The experimental systems support a requirement for these pathways in substantial components of the IL-4-conditioned state, including responses relevant to helminth immunity. They do not justify treating IRF4 as intrinsically reparative. In a different macrophage context, early MYC-driven glycolysis was reported to restrain IRF4 and thereby tune inflammatory cytokine output (20). Metabolism therefore does more than supply activated cells with energy. It changes the regulatory state in which IRF4 abundance and partner binding acquire meaning.

Temporal state is equally important. The IRF4 gene-regulatory module can integrate successive signals during T-helper differentiation, allowing earlier activation to alter later transcriptional responses (21). Dose-dependent changes in IRF4 also affected chromatin accessibility and peripheral Treg homeostasis in mice (16). These studies indicate that an IRF4-dependent phenotype can reflect the history of signalling and differentiation, not only the stimulus present when tissue is collected. Disease phase may consequently reverse the translational interpretation of a pathway. Inhibition during an inflammatory flare could attenuate an effector programme, whereas sustained inhibition during resolution might interfere with regulatory or repair functions. Direct comparisons of timing and withdrawal are sparse, so this proposition should guide experimental design rather than be presented as an established dosing rule.

The molecular evidence therefore supports a conditional rather than directional model of IRF4 activity (**Figure 1**). Cell lineage determines cofactor availability; receptor and cytokine inputs regulate abundance; phosphorylation and metabolism alter functional state; chromatin constrains accessible targets; and tissue and time assign phenotypic relevance. None of these variables acts as a universal switch. Their interaction explains why IRF4 can be causal in one compartment, associative in another and protective in a third. It also precludes a single numerical measure of IRF4 activity or inflammatory direction.

**Figure 1:**
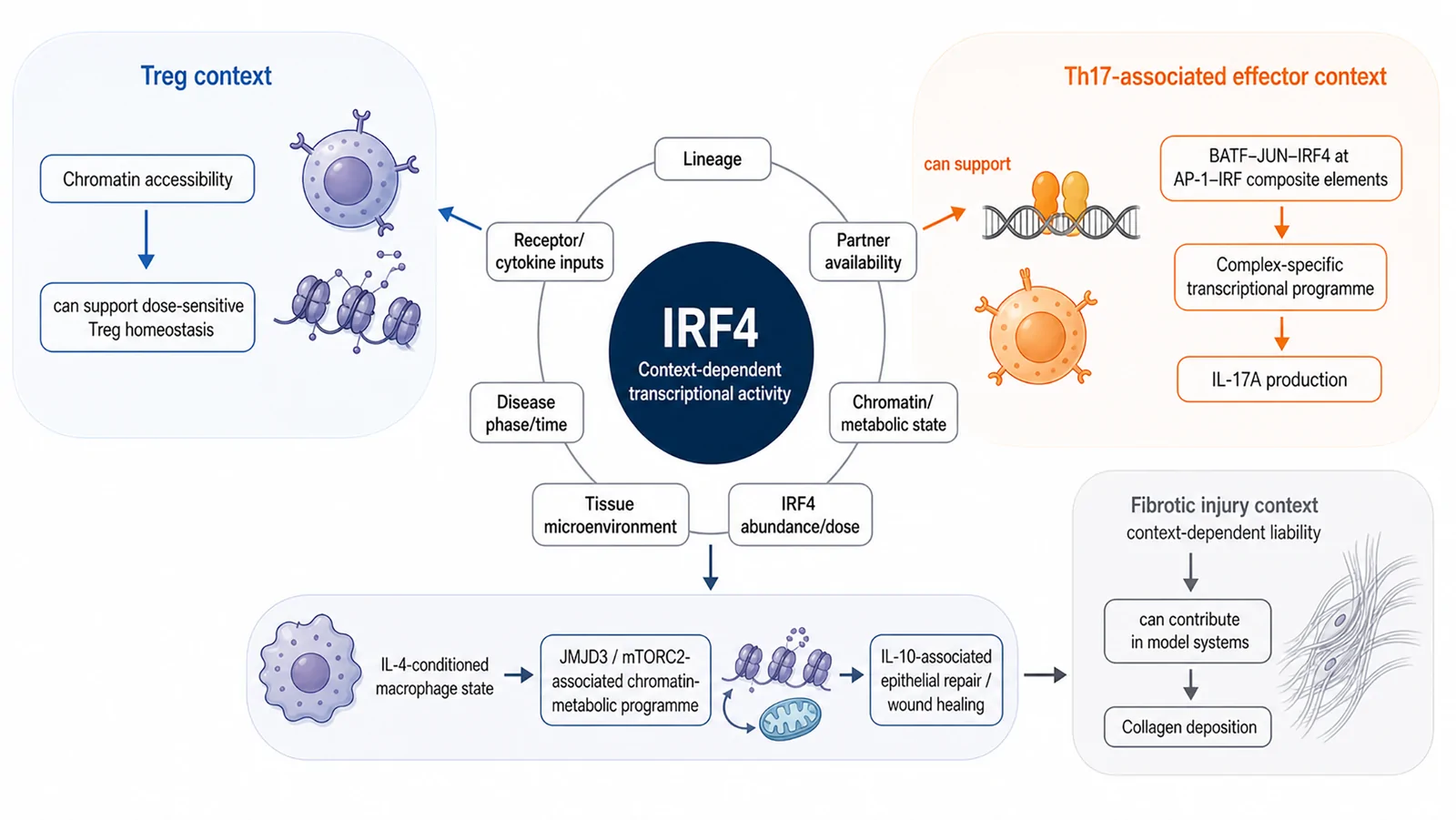
Context-dependent transcriptional activity of IRF4 across immune and tissue states. IRF4 output is shaped by lineage, receptor/cytokine inputs, partner availability, chromatin and metabolic state, IRF4 abundance/dose, tissue microenvironment, and disease phase/time. In activated effector T cells, BATF–JUN–IRF4 complexes acting at AP-1-IRF composite elements can support a Th17-associated transcriptional programme and IL-17A production. In regulatory T-cell contexts, IRF4 is linked to chromatin accessibility and dose-sensitive Treg homeostasis. In IL-4-conditioned macrophage contexts, JMJD3- and mTORC2-associated chromatin–metabolic programmes are linked to IL-10-associated epithelial repair and wound healing. By contrast, in specific fibrotic injury model systems, myeloid IRF4 activity can contribute to collagen deposition. The schematic represents context-dependent programme selection rather than a universal directional pathway or a validated therapeutic intervention map.

Measurement must reflect this complexity. IRF4 transcript abundance can locate a candidate cell population, and immunostaining can establish protein presence. Neither identifies the operative transcriptional complex or proves control of the disease phenotype. A stronger activity assessment combines protein abundance with relevant post-translational state, partner occupancy or complex-specific transcriptional output, chromatin accessibility, and a downstream molecular module. Disease causality then requires perturbation of IRF4 followed by concordant change in that module and in a disease-relevant phenotype. These distinctions are particularly important in human lesions, where altered cell composition may increase bulk IRF4 signals without increasing IRF4 activity within any individual cell type. These molecular determinants therefore supply both a mechanistic model and a rule for interpreting evidence.

This conditional model predicts non-equivalent outcomes across immune lineages. The relevant question is therefore not whether IRF4 is globally inflammatory, but which causal role its perturbation supports in each cell and tissue context.

## 4 Lineage-specific inflammatory circuits and biological liabilities

### 4.1 Dendritic cells: lineage specification, migration and polarisation

IRF4 has an established role in conventional dendritic-cell development and function, particularly in type 2 conventional dendritic cells (cDC2). Mouse genetic studies showed that IRF4 and IRF8 partition major dendritic-cell programmes. IRF4 was required for development or maintenance of CD11b-high, CD8α-negative populations, whereas IRF8 was associated more strongly with cDC1 biology (22, 23). IRF4-dependent dendritic cells also promoted Th2 differentiation in experimental systems (24, 25). In the intestine, related CD103-positive CD11b-positive dendritic-cell populations supported Th17 differentiation through cytokine programmes that included IL-6 (26). Human CD1c-positive dendritic cells share parts of this transcriptional and functional organisation, but the available comparison does not establish complete equivalence between human and mouse subsets (27). These findings establish IRF4 as a lineage and state regulator. Constitutive genetic dependence alone cannot determine how acute IRF4 reduction would affect an already differentiated human dendritic cell.

Skin-relevant evidence extends the lineage findings without closing the acute-causality gap. IRF4 supported migration of cutaneous dendritic cells to draining lymph nodes in mice during homeostasis and inflammation (28). A separate study linked CCR7- and IRF4-dependent dendritic cells to regulation of collecting lymphatic-vessel permeability (29). More recently, a PU.1/IRF4-dependent dendritic-cell programme was associated with retinoic-acid production and accelerated Treg development in an experimental anti-inflammatory setting (30). Together, these studies make dendritic-cell IRF4 a plausible regulator of antigen transport, polarisation, and tolerance, but they also expose competing outcomes of suppression. Reduced activity could attenuate pathogenic priming whilst impairing immune surveillance or regulatory-cell induction. The defensible classification is therefore an established lineage regulator and a context-specific circuit mediator in model systems. Direct evidence that acute, dendritic-cell-selective IRF4 inhibition treats human inflammatory skin disease remains absent.

### 4.2 Myeloid cells: state, tissue and phase beyond the M1/M2 shorthand

Myeloid studies illustrate why inflammatory direction should be assigned to a defined state rather than to IRF4 itself. IRF4 competed with IRF5 for MyD88 binding and reduced Toll-like receptor-induced inflammatory cytokine production in an experimental macrophage system (9). IL-4-conditioned macrophages used JMJD3–IRF4 and mTORC2–IRF4 programmes, and myeloid IRF4 limited adipose inflammation and insulin resistance in mice (11, 19, 31). These contexts support inflammation-limiting or type 2-associated functions. A different pattern appeared after severe renal ischaemic injury: deletion of myeloid Irf4 reduced macrophage recruitment and activation and protected mice from kidney fibrosis (12). IRF4-linked macrophage programmes also contributed to silica-induced pulmonary fibrosis (13). In microglial/LPS systems, BATF2–IRF4–STAT circuitry provided another pro-inflammatory example, although the compound used in that work was not an IRF4-selective inhibitor (32). Organ, stimulus, lineage composition, and disease phase changed together across these studies, so their effects cannot be ranked on one pro- versus anti-inflammatory axis. Although “M1/M2” is used in parts of the cited literature, it is only a historical shorthand here and does not adequately represent the heterogeneous, tissue- and time-dependent macrophage states discussed below. The historical M1/M2 labels are used only as shorthand for experimental polarisation conditions; they do not capture the continuous, tissue- and time-dependent macrophage states now recognised *in vivo*.

Repair evidence prevents fibrosis studies from being generalised into a systemic-inhibition rationale. In a reductionist epithelial-recovery model, IRF4-dependent macrophage IL-10 production was associated with improved epithelial repair (14). More directly relevant to skin, a 2026 mouse study linked an IRF4-dependent monocyte-derived antigen-presenting-cell programme to IL-27, Treg responses, and wound healing, with impairment in diabetic animals (15). This recent report supports necessity in the tested wound context, not a replicated human repair mechanism. It also leaves open whether the phenotype reflects cell-intrinsic IRF4 activity, changes in monocyte-derived cell composition, or both. The combined myeloid evidence supports a context- and phase-dependent mediator classification. Adult-inducible perturbation within the same tissue, applied at inflammatory and repair phases, is needed to distinguish an acute mediator effect from developmental or compositional change. Binary M1/M2 terminology obscures that question because it compresses distinct transcriptional states and functions into labels whose meaning varies across models.

### 4.3 T cells: effector potency, regulation and dose-dependent risk

IRF4 is necessary for several T-cell effector programmes in model systems. T-cell receptor signal strength regulates IRF4 abundance, whilst BATF-JUN cooperation enables access to effector-associated regulatory elements (2, 6). Conditional or genetic studies linked IRF4 to CD8-positive T-cell clonal expansion and effector differentiation (4, 5). Within CD4-positive cells, IRF4 was required for Th17 and Th9 differentiation and participated in a dynamic module coordinating T-helper-cell fate (7, 8, 21). It also contributes to T follicular helper differentiation and cooperates with BLIMP1 in effector Treg programmes (33, 34). FLI1-IRF4 cooperation further refined the Th17 programme in murine polarised cells (17). However, adult perturbation studies disagree about maintenance after differentiation. Induced Irf4 deletion in infection models impaired intestinal accumulation more strongly than established Th2 or Th17 cytokine competence (35), whereas a 2026 ex vivo study reported that both conventional and pathogenic differentiated Th17 cells remained IRF4-dependent (36). Differences in tissue, activation history, deletion timing, and the operational definition of maintenance may explain the divergence. This unresolved boundary is directly relevant to therapy because established disease contains differentiated pathogenic cells, not only naive precursors.

Regulatory and protective functions set exposure-dependent boundaries around the effector evidence. Dose-sensitive IRF4 altered chromatin accessibility and peripheral Treg homeostasis in mice (16). IRF4 also sustained protective CD8 responses in infection models and supported antitumour CD8 activity (4, 37). Human genetic evidence strengthens the conclusion that IRF4 has pleiotropic immune functions: inherited IRF4 dysfunction caused a multisystem immunodeficiency involving several immune compartments (38). Germline disease, however, is not a dose-matched model of transient adult inhibition. It includes developmental effects and lifelong exposure that may overstate some pharmacological liabilities whilst failing to predict others. These studies are best used as potential safety liabilities. They identify Treg stability, protective cellular immunity and tumour surveillance as domains requiring exposure-matched testing. They neither provide clinical incidence estimates nor prove that a useful therapeutic window cannot exist.

The possibility that a high-IRF4 pathogenic effector programme could be more sensitive to partial inhibition than a protective compartment remains testable. Evidence for graded IRF4 function makes the hypothesis mechanistically coherent, but the required comparison has not been completed with one intervention, exposure range, and recovery design. The conflicting maintenance studies (35, 36) also caution that sensitivity may change with tissue residence and activation history. A therapeutic-window claim therefore requires simultaneous measurement of pathogenic effector suppression, regulatory-cell stability, host-defence function, tissue trafficking, and reversibility after withdrawal. Until then, T-cell evidence supports IRF4 as an effector-lineage integrator and necessary mediator in selected models, alongside protective functions that constrain systemic targeting.

### 4.4 B cells, plasma cells and ILC3s: humoral and barrier-defence boundaries

IRF4 controls successive B-cell fate decisions through abundance- and context-dependent transcriptional programmes. Genetic and systems studies linked IRF4 to class-switch recombination, germinal-centre exit, and plasma-cell differentiation, including induction of PRDM1/BLIMP1 at higher IRF4 abundance (3, 39, 40). Recent work showed that stimulation history is recorded through progressively accessible IRF4-controlled plasma-cell loci and the BLIMP1-BACH2 balance, influencing memory B-cell fate on recall (41). TLR-NF-kappaB-IRF4 signalling also supported protective extrafollicular responses, whilst IFN-alpha/TLR9/mTOR signalling sustained IRF4 and plasma-cell differentiation (42, 43). Conversely, restricting IRF4 hyperactivation ameliorated murine lupus (44), and a 2025 IRF4 K123R model altered B-cell state and reduced lupus whilst increasing B7-H1 (45). These studies show that pathological and protective B-cell programmes can both depend on IRF4, and that changing IRF4 function is not equivalent to reducing IRF4 abundance.

Barrier defence broadens the liability map beyond lymphocytes conventionally emphasised in skin inflammation. A 2025 conditional and chromatin study identified IRF4-dependent regulation of the homeostasis and function of intestinal group 3 innate lymphoid cells (ILC3s) (46). The evidence is direct for the tested gut lineage but indirect to cutaneous efficacy. Its value here is not to expand a catalogue of IRF4-expressing cells. It shows that systemic exposure could affect protective compartments outside the target organ, and that cross-tissue studies should inform safety testing without being presented as skin-disease evidence.

Across dendritic, myeloid, T-cell, B-cell, plasma-cell, and ILC3 compartments, IRF4 most consistently appears as a lineage- and state-dependent regulator. It meets the narrower mediator criterion only where same-context perturbation links an IRF4 programme to a defined molecular output and phenotype. The same map identifies non-commensurate liabilities in migration, tissue repair, immune regulation, antibody production, barrier defence, and cellular immunity. These findings do not establish a quantified clinical risk, but they determine what a credible translational programme must measure (**Table 1**).

**Table 1:** Lineage-specific IRF4 roles, causal classifications and translational liabilities.

Lineage/tissue	Perturbation/design	Principal IRF4-dependent output	Evidence basis	Current causal classification	Directness to skin	Translational boundary
cDC2/skin and mucosa	Germline or conditional genetics; migration and vessel assays	cDC2 differentiation, cutaneous migration, type 2/type 17 support	Mouse genetics; human-mouse comparison (22–29)	Established lineage regulator; plausible circuit mediator	Direct for cutaneous migration; indirect for disease efficacy	Inhibition could alter antigen transport, priming and lymphatic-vessel permeability
Myeloid cells	Knockout, signalling, metabolic and injury models	TLR restraint, alternative activation, fibrosis or repair	Genetic and mechanistic models across organs (9, 11–15, 19)	Context- and phase-dependent mediator	Direct only for the wound-repair model	Direction cannot be generalised across organs or disease phases
Effector and regulatory T cells	Conditional, graded and molecular perturbation	Th9, Th17, Tfh, Treg and CD8 programmes	Graded, conditional and molecular perturbation (2, 4–8, 16, 17, 33, 34, 37, 38)	Strong model-system necessity for defined states	Mostly indirect; psoriasis study tested a disease-linked CD4-positive programme	Systemic inhibition may affect tolerance, host defence and tumour immunity
B/plasma cells	Genetic, systems and infection perturbation	Class switching, plasma-cell differentiation and extrafollicular defence	Genetic, systems and infection models (3, 39, 40, 42, 43)	Established fate regulator	Indirect to skin	Durable inhibition may compromise humoral immunity
Intestinal ILC3s	Conditional deletion and chromatin analysis	Barrier and host-defence programmes	Conditional and chromatin evidence (46)	Protective lineage regulator in gut	Indirect to skin	Cross-tissue safety signal, not direct skin-disease evidence

Lineage evidence supplies mechanistic priors and defines safety boundaries. Disease-specific skin evidence must nevertheless be evaluated independently to determine whether IRF4 crosses from biological plausibility to causal and therapeutically actionable support.

### 4.5 Epidermal and structural-cell context

Evidence from skin-resident structural cells offers an additional, non-immune perspective on IRF4 function. In melanocytes, the intronic variant rs12203592 regulates IRF4 expression through an enhancer mechanism and influences tyrosinase-dependent pigmentation (47, 48). The same variant has also been associated with cutaneous squamous cell carcinoma risk in organ-transplant recipients (49). These findings show that IRF4 can have biologically relevant functions outside immune cells; however, they do not establish an IRF4-dependent inflammatory mechanism in keratinocytes, melanocytes or fibroblasts in psoriasis or atopic dermatitis. Direct, cell-specific perturbation studies in inflammatory skin disease are still needed.

This review is centred on immune-lineage evidence, but keratinocytes are active participants in psoriatic inflammation rather than passive targets. Cytokine-stimulated keratinocytes can release antimicrobial peptides, cytokines and chemokines that reinforce local immune-cell recruitment and inflammatory feedback loops (50). The reported NLRP3–IRF4–IL-33 mechanism provides an epithelial entry point in atopic dermatitis, but it does not establish that IRF4 independently drives a keratinocyte inflammatory circuit in psoriasis or AD (51, 52). Evidence directly testing IRF4 function in keratinocytes, melanocytes or fibroblasts in inflammatory skin disease remains limited. We therefore treat these resident structural cells as an important evidence gap, rather than extending immune-cell findings to them by inference.

## 5 IRF4 in inflammatory skin disease: evidence by compartment

The lineage studies discussed above establish mechanisms through which IRF4 can shape cutaneous inflammation, but they do not by themselves demonstrate disease-specific causality. The distinction is important because increased IRF4 expression may reflect immune-cell recruitment, differentiation, or activation rather than a reversible pathogenic dependency. This section therefore separates human observations from same-context mechanistic perturbation, animal genetics, and pharmacological intervention. Under this framework, psoriasis provides the most developed disease-linked preclinical hypothesis, whereas the evidence in atopic dermatitis (AD) and inflammatory overlap states remains earlier or indirect. Skin wound repair provides a complementary test of the model by showing that IRF4-dependent circuitry can also support tissue restoration.

### 5.1 Psoriasis: the most developed disease-linked preclinical hypothesis

The present psoriasis discussion is intentionally proportionate to the evidence base: its detailed analysis reflects the breadth of measurements within one 2025 study, not independent replication. This distinction is made throughout the section and in the conclusion. Future work should test whether IRF4-related activity is supported by independent genetic, spatial or single-cell evidence and whether it persists across treatment-exposed lesions.

To broaden the disease-context evidence beyond the single IRF4-targeting study, we considered large psoriasis genetic and cell-resolved datasets. A 2025 GWAS meta-analysis integrated psoriasis susceptibility signals with single-cell expression profiles in diseased skin, but did not establish an IRF4-proximal psoriasis susceptibility signal (53). Separately, single-cell and spatial RNA sequencing identified marked keratinocyte-state changes and inflammatory fibroblast-keratinocyte interactions in psoriatic skin (54). These studies define relevant cellular contexts for testing IRF4-associated programmes; they do not replace direct, cell-specific IRF4 perturbation or independent replication.

#### 5.1.1 Human tissue and cellular evidence

The principal disease-focused evidence comes from a 2025 study that examined IRF4 across human samples and experimental models (55). IRF4 activity and expression were increased in psoriatic lesions, and the study linked this signal to the IL-23A- and IL-1beta-rich inflammatory environment. IRF4 was also associated with circulating and cutaneous T helper 1 and Th17 responses. These findings provide a human disease anchor, but lesion-level enrichment cannot determine whether IRF4 initiates inflammation, amplifies an established cytokine circuit, or marks activated and recruited lymphocytes. Spatial transcriptomics has shown that psoriatic severity is organised across distinct epidermal, macrophage-, fibroblast-, and lymphatic-enriched tissue regions (56), reinforcing the risk of interpreting bulk abundance as cell-intrinsic activity. The human observations therefore provide tissue association and pathway concordance rather than therapeutic evidence.

The study nevertheless went beyond static lesion profiling. CD4-positive T cells from the peripheral blood of patients with severe psoriasis were exposed ex vivo to IRF4-directed antisense oligonucleotides (ASOs). Investigators then assessed the proposed downstream programme (55). This experiment moves the evidence closer to human perturbation, but it remains an isolated-cell assay outside the lesional niche. It does not reproduce interactions amongst keratinocytes, dendritic cells, resident lymphocytes, stromal cells, and recruited myeloid cells, nor does it establish delivery, exposure, or clinical response in patients. Human lesional cultures or organotypic systems with cell-resolved target engagement would provide a more stringent bridge between this cellular result and the disease.

#### 5.1.2 Mechanistic evidence

The proposed cascade connects upstream inflammatory cytokines to a CD4-positive T-cell transcriptional programme. In the reported systems, IL-1β and IL-23A increased IRF4, which bound the *EP300* promoter and enhanced its transcription. EP300 activity was then linked to RORγt/*RORC* and IL-17A output, defining an IL-1β/IL-23A–IRF4–EP300–RORC–IL-17A pathway (55). Chromatin immunoprecipitation, promoter-reporter experiments, expression perturbation, ASO screening, and recovery experiments supported several steps in this sequence. The mechanism is compatible with established IRF4 dependence of Th17 differentiation and with cooperative IRF4 transcriptional programmes in activated T cells (2, 7, 17). These supporting studies establish biological plausibility, however, not independent replication of the complete psoriasis pathway.

Because the experiments span promoter assays, murine lesional cells, primary mouse T cells, and circulating patient T cells, they do not constitute a continuous human-lesion pathway. Promoter assays, murine lesional CD4-positive T cells, primary mouse T cells, and patient-derived circulating CD4-positive T cells answer different questions. Together, they support a model in which IRF4 contributes to an IL-17A-producing effector programme. They do not show that every step operates with the same strength in each human lesional cell type. Rescue with IRF4 overexpression supports the interpretation that ASO effects were linked to IRF4 reduction. Orthogonal perturbations and independent replication are still needed to exclude sequence-specific or context-specific effects. The most defensible mechanistic classification is a downstream amplifier and necessary mediator in the tested cellular systems, not a sufficient driver of human psoriasis.

#### 5.1.3 Genetic and ASO intervention evidence in mice

Genetic experiments supplied a disease-relevant perturbation test. Both systemic *Irf4* deletion and CD4-lineage conditional deletion attenuated imiquimod (IMQ)-induced psoriasiform dermatitis, with concordant reductions in IL-17A-producing CD4-positive T cells and components of the proposed downstream programme (55). The conditional model improves cell attribution by implicating CD4-positive T cells, whilst the systemic model captures effects across multiple IRF4-dependent compartments. Neither design is equivalent to reversible inhibition in an immunologically mature animal. Developmental changes in T-cell differentiation and immune composition can contribute to the phenotype, and loss of IRF4 in other lineages may produce additional effects in the systemic knockout.

The ASO experiments partly address this limitation because they tested an acquired intervention rather than inherited absence. Candidate ASOs were screened in primary T cells, and treatment reduced IRF4 and downstream EP300/RORγt/IL-17A readouts. In mice, ASO administration began before IMQ exposure: animals received weekly subcutaneous dosing for four weeks, and IMQ was initiated during the final dosing week (55). The resulting reduction in psoriasiform features and IL-17A-producing cutaneous CD4-positive T cells therefore provides pre-induction or prophylactic proof of concept, not evidence that IRF4 reduction reverses established dermatitis. “Pharmacological validation” would be too strong. The study did not establish successful skin-restricted exposure, relative uptake amongst pathogenic and protective cell populations, or a therapeutic index. Systemic immune effects could contribute to improvement, and oligonucleotide chemistry can introduce delivery and innate-sensing variables that require sequence controls, orthogonal ASOs, biodistribution measurements, and exposure-response analysis.

Across four evidence compartments, the psoriasis case forms an internally coherent but single-study preclinical package (**Figure 2**). Human tissue and ex vivo cell data provide disease relevance, whereas mechanistic studies connect IRF4 to an EP300-RORγt-IL-17A programme. Genetic models support necessity, and pre-induction ASO treatment provides an intervention benchmark (55). Confidence is limited because these findings are concentrated in one study and use prophylactic dosing in an acute IMQ model. This model does not reproduce the chronic, relapsing and systemic biology of human psoriasis. The responding cells, efficacy in established disease, adult reversibility, lesional pharmacokinetics and pharmacodynamics, toxicity, durability after withdrawal and effects on regulatory T cells and host defence remain unresolved. IRF4 is therefore best classified as a necessary mediator of induction or early expression in the reported psoriasiform systems. It remains the most developed disease-linked preclinical hypothesis identified in the accessible searches. It is not yet a validated human psoriasis target.

**Figure 2:**
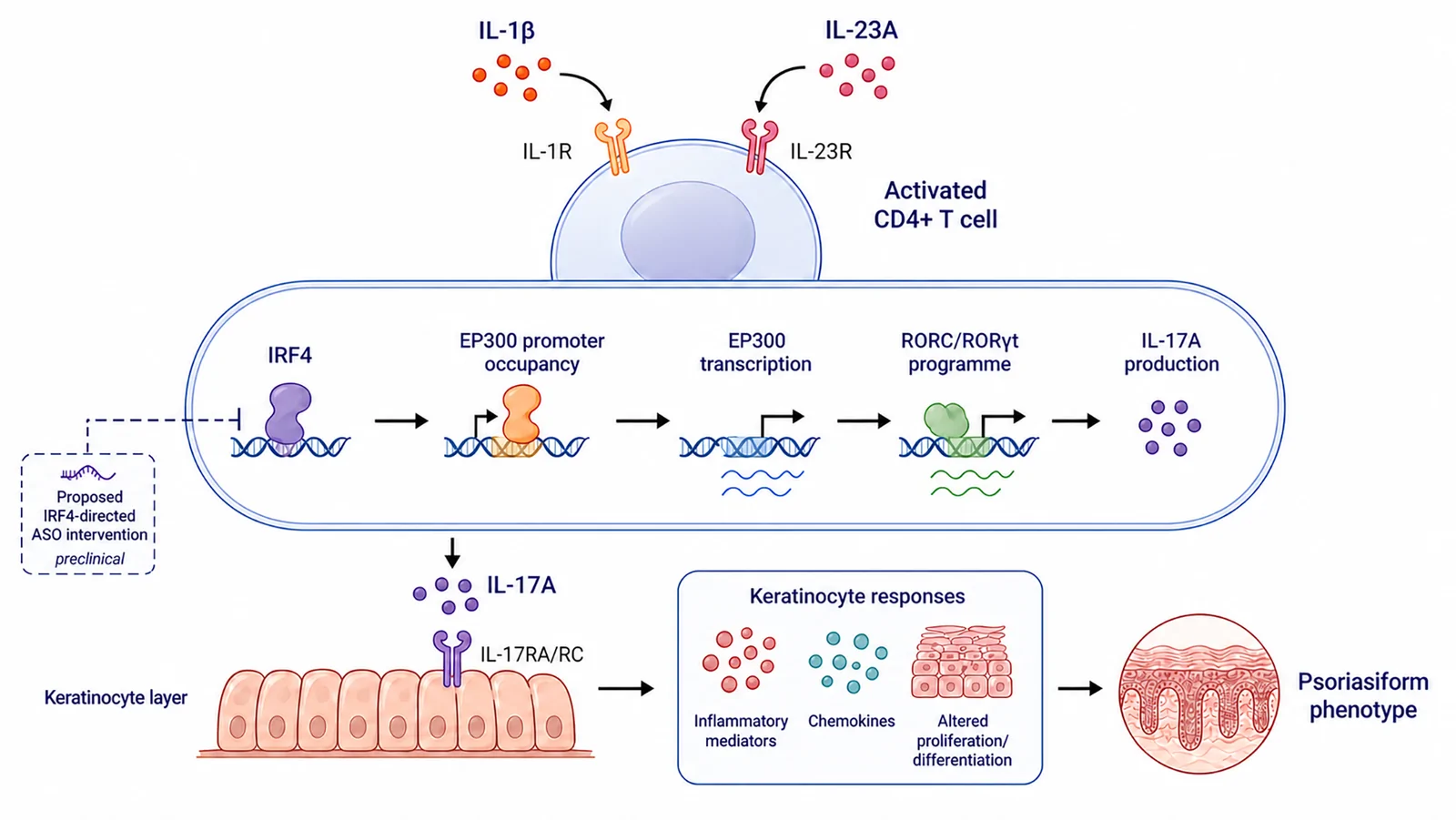
Evidence map for IRF4 in inflammatory skin disease. The psoriasis evidence package spans lesion association, ex vivo perturbation and pre-induction mouse studies, but decisive human-lesion, pharmacokinetic/pharmacodynamic, reversibility and safety evidence remains unavailable. The diagram is a synthesis of the reviewed evidence, not a validated continuous mechanism in human lesions.

### 5.2 Atopic dermatitis: a candidate epithelial and immune-network amplifier

IRF4 has two conceptually distinct links to AD: an immune-network rationale derived largely from dendritic-cell and T-helper biology, and a reported epithelial mechanism involving IL-33. IRF4-dependent conventional type 2 dendritic-cell programmes can promote T-helper 2 (Th2) differentiation in mouse systems (24, 25). More recently, mechanical skin injury was shown to induce basophil-dependent IL-4, which acted on skin-derived dendritic cells to increase their Th2-polarising capacity and promote allergic skin inflammation in mice (57). The latter study did not perturb IRF4. It supports an IL-4-responsive cutaneous dendritic-cell circuit in which prior lineage studies make IRF4 biologically plausible. However, it cannot establish IRF4 as the causal intermediate or as an incremental target.

The epithelial evidence is more disease proximal. Zheng and colleagues reported increased NLRP3 and IL33 expression in AD lesions and examined an inflammasome-independent nuclear function of NLRP3 in epithelial cells (51). In immortalised epithelial cells, IRF4 perturbation altered IL-33 expression, and NLRP3 physically interacted with IRF4 under the reported stimulation conditions. The DNA-binding evidence assigned direct recognition of the IL33 promoter to NLRP3 rather than IRF4. NLRP3 deficiency also reduced IL-33 and epidermal inflammation in the MC903 mouse model, but the *in vivo* genetic test targeted Nlrp3, not Irf4 (51). The article was corrected in 2024 after the publisher identified and replaced a duplicated IRF4 immunoblot panel (52). This image-integrity error lowers confidence in the expression component and reinforces the need for independent replication. However, the reported IRF4 overexpression, small interfering RNA, interaction and NLRP3 DNA-binding experiments remain separate lines of evidence. The study therefore supports IRF4 as a partner in a candidate epithelial transcriptional complex, but it does not establish that epithelial IRF4 is independently necessary for the complete AD-like phenotype.

These epithelial and immune observations form a coherent hypothesis but not a validated disease dependency. Independent replication, spatial localisation of the NLRP3-IRF4 complex in human lesions, adult epithelial- and immune-lineage perturbation, and endotype-resolved activity measurements are missing. Comparative experiments are also needed to determine whether reducing IRF4 offers information or efficacy beyond IL-4Rα or IL-13 blockade and JAK inhibition. IRF4 is therefore classified as a candidate epithelial and immune-network amplifier in AD. Human clinical causality, treatment prediction, and added therapeutic value remain unvalidated.

### 5.3 Cutaneous overlap states and wound repair: relevance without direct efficacy

Systemic autoimmune and inflammatory models broaden the mechanistic context but provide little direct support for cutaneous IRF4 inhibition. In experimental Sjögren syndrome, artesunate reduced IRF4-associated glycolysis and Th17 responses and improved disease-related outcomes (58). This result indicates that an IRF4-linked metabolic-effector programme can be modulated, but artesunate has multiple biological actions and should not be described as an IRF4-selective inhibitor. Repression of IRF4 hyperactivation also restricted disease in a murine lupus model (44), whereas IRF4 deficiency reduced acute graft-versus-host disease-associated colitis in another model (59). The tissues, initiating insults, immune populations, and interventions differ from those in inflammatory dermatoses. Shared Th17 or B-cell biology can generate testable hypotheses, but it does not justify extrapolating efficacy to cutaneous lupus, Sjögren-associated skin disease, or cutaneous graft-versus-host disease.

Wound repair provides a skin-specific counterweight to such extrapolation. In a recent mouse study, monocyte-lineage *Irf4* loss impaired differentiation of wound monocytes into antigen-presenting cells, reduced IL-27 and activated regulatory T cells, and delayed repair (15). Adoptive transfer of IRF4-expressing monocytes or local IL-27 restored elements of the cellular pathway and improved healing, strengthening the causal link within that model (15). The circuit was also impaired in diabetic wounds. Although this single recent study requires replication and does not quantify the effect of partial, transient inhibition, it demonstrates that IRF4 activity in injured skin need not be pathogenic. The same target may contribute to inflammatory amplification during one disease phase and to immune regulation and tissue restoration during another.

The overlap and repair evidence therefore changes the translational question. It is insufficient to ask whether IRF4 reduction suppresses a lesion at peak inflammation. Studies must determine what happens during resolution, barrier restoration, infection, and re-injury, and whether effects in the skin remain separable from blood and lymphoid compartments. Paired blood-skin profiling, spatial localisation of active IRF4 complexes, adult cell-restricted perturbation, and longitudinal sampling across flare and repair could distinguish disease mediation from systemic or phase-specific effects. Until these experiments are available, overlap-state studies remain cross-tissue pathway evidence, whilst wound healing defines a protective cutaneous liability for any sustained IRF4-directed strategy (**Table 2**).

**Table 2:** Causal-discrimination matrix for inflammatory skin evidence.

Condition	Human evidence	Model/evidence compartment	Same-context perturbation	Strongest alternative explanation	Current classification	Decisive missing evidence
Psoriasis	Lesional association and disease-linked cellular observations in one 2025 study (55)	Human lesions and ex vivo cells; acute IMQ mouse model	Mechanistic experiments, genetic perturbation and IRF4 ASO (55)	Study-specific, developmental or IMQ-model effects; non-sequence delivery effects	Most developed disease-linked preclinical hypothesis; necessary mediator in reported systems	Independent human perturbation, responding-cell attribution, skin PK/PD, toxicology, reversibility and therapeutic window
Atopic dermatitis	Epithelial AD context for the NLRP3-IRF4-IL-33 mechanism (51, 52)	AD lesions, immortalised epithelial cells and MC903 mice	IRF4-related molecular perturbation in epithelial cells; the *in vivo* deletion targeted NLRP3 (51)	IRF4 may be a complex partner or downstream marker; one corrected blot reduces confidence in the expression component	Candidate epithelial and immune-network amplifier	Independent replication, cell-resolved human activity, endotype association and intervention evidence
Skin wound repair	No human IRF4 perturbation identified	Diabetic and non-diabetic mouse wounds	IRF4-dependent monocyte-derived APC-Treg axis with adoptive-transfer and IL-27 rescue (15)	Developmental or model-specific repair effects	Protective cutaneous counterweight in one mouse setting	Independent replication, human validation, exposure-response, timing and reversibility
Cutaneous overlap states	Predominantly systemic or cross-tissue relevance	Murine lupus, Sjögren syndrome and aGVHD-colitis models	Pathway-level genetic or pharmacological perturbation (44, 58, 59)	Shared systemic pathways may not operate in cutaneous lesions	Indirect to cutaneous efficacy	Paired blood-skin perturbation and disease-specific target engagement
Epidermal/structural-cell context	Keratinocyte inflammatory feedback and limited reported NLRP3–IRF4–IL-33 epithelial evidence; melanocyte and fibroblast data are indirect to inflammatory disease	Psoriasis and AD context; direct IRF4 perturbation evidence remains limited	No same-context evidence establishing IRF4 as an independent driver	IRF4 may be a partner, marker or context-specific mediator rather than an independent driver	Evidence gap; immune-cell findings should not be generalised to resident structural cells	Cell-resolved human expression, same-context perturbation and independent replication

The matrix is a decision aid rather than a ranking of diseases. Each row identifies a distinct evidence compartment, a plausible alternative explanation and an experiment capable of changing the present classification.

## 6 From biological causality to therapeutic tractability

### 6.1 Direct reduction and local delivery

Amongst the inflammatory studies reviewed, direct IRF4 reduction has reached proof of concept only in preclinical models. In an imiquimod-induced mouse model of psoriasiform inflammation, an IRF4-targeting antisense oligonucleotide (ASO) reduced the reported inflammatory phenotype and associated pathway outputs (55). This intervention is more disease-proximal than studies in which an upstream kinase, cytokine, or metabolic pathway was altered, because IRF4 itself was the intended molecular target. The result nevertheless remains an animal pharmacological intervention within a single evidence package. It does not establish efficacy in human psoriasis or identify the responding cell population. Nor does it show that benefit arose exclusively from sequence-specific IRF4 reduction rather than chemistry- or delivery-related effects.

Skin accessibility creates an experimental advantage, but it does not solve delivery. Development of a topical, intradermal or otherwise skin-directed oligonucleotide requires measurement of lesional and plasma exposure, cellular uptake, endosomal escape and IRF4 reduction. The duration of silencing and recovery after treatment withdrawal must also be defined. Chemistry-matched controls should also distinguish sequence-specific activity from innate immune sensing of the oligonucleotide. Serial skin sampling could link local exposure to an IRF4-dependent transcriptional response, but no validated human IRF4-directed skin-delivery or pharmacokinetic and pharmacodynamic programme was identified in the accessible searches. Local delivery offers a test of compartment selectivity; successful restriction and safety have not yet been shown.

### 6.2 Complex-, dose- and time-selective strategies

The molecular organisation of IRF4 provides several hypotheses for reducing pathogenic activity without suppressing the whole protein in every compartment. IRF4 cooperates with BATF-JUN proteins in activated T cells, and graded IRF4 abundance changes effector T-cell differentiation (2, 6). More recent work has linked FLI1-IRF4 cooperation to murine Th17 programmes and dose-dependent IRF4 activity to Treg homeostasis and chromatin accessibility (16, 17). A function-altering IRF4 K123R model in B cells also provides proof of principle that qualitative modulation can produce an outcome distinct from whole-protein loss (45). These observations justify tests of partner-, enhancer-, dose-, or schedule-selective perturbation. They do not show that an IRF4 interface is druggable, that a pathogenic complex can be inhibited independently of protective complexes, or that network compensation will remain limited.

Candidate modalities include small interfering RNA, targeted protein degradation, and molecules designed to disrupt a defined IRF4-containing complex. Each remains a development hypothesis in inflammatory disease. A valid complex-selectivity experiment must demonstrate engagement of the intended interface and loss of the corresponding chromatin occupancy or transcriptional module. It must also preserve a predefined protective programme whilst producing a disease-relevant phenotypic effect. Rescue experiments and perturbation sequencing could test whether the phenotype is attributable to the proposed complex rather than generalised T-cell dysfunction. In particular, disruption of BATF-JUN-IRF4 cannot be assumed to spare ETS-family-dependent B-cell functions. That separation must be measured.

Exposure schedule may be as important as molecular selectivity. Transient partial suppression during an inflammatory flare could produce a different biological result from sustained, near-complete systemic depletion. Graded IRF4 effects in effector and regulatory T cells make knockout phenotypes poor surrogates for all clinically relevant exposures (6, 16). Dose-ranging studies can connect IRF4 target engagement to pathogenic-circuit suppression and recovery of protective functions after withdrawal. A treatment window exists only if these measurements separate at clinically plausible exposures.

### 6.3 Indirect modulation and attribution

Upstream interventions can alter IRF4-dependent programmes without being IRF4-selective therapies. ROCK2-mediated phosphorylation contributes to IRF4-dependent IL-17 and IL-21 production, whilst JMJD3 and mTORC2 regulate IRF4-associated macrophage programmes (11, 18, 19). Artesunate reduced IRF4-associated glycolysis and T helper 17 responses in an experimental Sjögren syndrome setting, but its activity cannot be reduced to selective IRF4 inhibition (58). The same attribution rule applies to JAK inhibitors, cytokine biologics, metabolic interventions and epigenetic agents. A clinical or animal response identifies IRF4 as mediator only if target engagement and IRF4 dependency are demonstrated.

Indirect modulation may still be therapeutically sufficient. This creates a demanding comparator for direct IRF4 development because established IL-17/IL-23 therapies already control psoriasis effectively, and biologics or JAK inhibitors provide multiple targeted options in AD (60, 61). A direct approach should therefore be compared with relevant upstream blockade in the same model or patient-derived system. Progression would be justified only if IRF4 perturbation adds mechanistic or phenotypic value, such as suppressing a residual pathogenic module, restoring activity in a pathway-resistant state, or improving local control. Mediation analysis, orthogonal perturbations, and genetic rescue should separate an IRF4-dependent response from a parallel consequence of upstream inhibition.

### 6.4 Biomarkers, safety boundaries and development gates

Target engagement must be defined at the level of the pathogenic IRF4 programme. IRF4 transcript abundance alone is inadequate because protein amount, post-translational regulation, partner occupancy, and chromatin accessibility determine output. In psoriasis, the reported IRF4-EP300-RORC and IL-17A module is a candidate pharmacodynamic signature, not a validated biomarker (55). A biomarker-led study would combine cell-resolved IRF4 protein or activity, a partner-complex transcriptional signature, spatial localisation within a pathogenic niche, and an early on-treatment change that precedes clinical improvement. Blood measurements are useful for quantifying systemic spillover, not as substitutes for lesional target engagement.

Protective IRF4-dependent functions define safety-monitoring domains that require exposure-matched testing. Rare human IRF4 dysfunction affects multiple immune compartments, but lifelong genetic disease does not model transient adult inhibition (38). Mouse and ex vivo studies implicate IRF4 in regulatory T-cell homeostasis, B-cell responses, dendritic-cell migration and lymphatic-vessel permeability, antitumour CD8 T-cell function, and wound repair (15, 16, 28, 29, 37, 42). These studies do not quantify clinical adverse-event rates. They identify what should be monitored: infection, vaccine responses, immunoglobulin levels, wound closure, tumour-surveillance proxies, and durable changes in immune-cell compartments.

Clinical progression requires reproducible perturbational evidence in human tissue, attribution to the relevant cell population and measurable, reversible target engagement. It also requires efficacy across multiple disease-relevant models and an acceptable margin between disease modulation and protective immune dysfunction. Failure to reproduce the phenotype with an orthogonal IRF4 reagent, absence of downstream pathway change despite target engagement, or non-recovery of protective functions after withdrawal should stop or redirect development. Biological causality is necessary, but it is not sufficient for therapeutic tractability.

The comparative target-product decision should be explicit. Direct IRF4 development is justified only if it adds value beyond established pathway control (60, 61). One example would be an IRF4-defined pathogenic module that persists despite IL-23/IL-17 blockade in psoriasis or IL-4Ralpha/IL-13 blockade and JAK inhibition in AD. A go decision would require concordant target engagement and phenotypic benefit at local or systemic exposures that preserve prespecified Treg, B-cell, CD8 T-cell, intestinal ILC3, and repair functions. Development should stop if required exposures cause durable immune dysfunction, if an orthogonal reagent fails to reproduce the response, or if a safer upstream intervention fully explains the benefit. The relevant product concept is not an IRF4 inhibitor in the abstract. It is a route-, dose-, schedule-, cell-, and biomarker-defined intervention that separates pathogenic from protective IRF4 programmes.

## 7 Discussion: evidence gaps and a falsifiable research agenda

Human disease causality is the first unresolved gate. Perturbing patient-derived psoriatic and atopic lesional cultures, organotypic skin, or primary cell systems with at least two orthogonal IRF4 reagents would address this gate. Concordant change in a prespecified downstream module and disease-relevant inflammatory phenotype would support acute mediation. In psoriasis, the reported IRF4-EP300-RORC-IL-17A programme provides one candidate readout (55). In atopic dermatitis, epithelial IL-33 regulation and immune-cell type 2 programmes require separate tests rather than assumed to form one continuous axis (51–57). Efficient IRF4 reduction without the predicted molecular or phenotypic response would weaken the claim that IRF4 is an acute disease mediator in that context.

Cell attribution requires perturbation, not expression mapping alone. Adult-inducible conditional models, cell-targeted delivery, spatial target-engagement measurements, and single-cell multi-omics can distinguish T-cell, dendritic-cell, epithelial, and myeloid contributions. Multi-omics can locate the response and define its molecular state, whereas causality still requires intervention. If disease improvement occurs whilst the proposed pathogenic population remains unperturbed, the mechanism would require revision. Conversely, rescue of the downstream programme or phenotype by restoring IRF4 in a defined population would strengthen cell-specific attribution and reduce developmental confounding.

The psoriasis hypothesis also requires independent replication. The present evidence is concentrated predominantly in one 2025 study that combined human-lesion observations, mechanistic experiments, mouse genetics, and ASO treatment (55). Independent replication requires orthogonal reagents, genetic rescue, more than one formulation, and chronic or relapsing models. Treatment-exposed disease belongs in human lesional systems because prior IL-23, IL-17, or JAK-pathway therapy may alter the cellular niche in which IRF4 operates. Failure of an orthogonal perturbation to reproduce the molecular and phenotypic effects would challenge the current causal chain rather than merely indicate model variability.

Dose, timing, and reversibility are most informative within the same efficacy-safety experiment. Comparison of partial and near-complete inhibition is needed during disease initiation, established inflammation, resolution, and repair. Withdrawal studies can measure restoration of the intended immune compartments and functions. The IRF4-dependent monocyte-derived antigen-presenting-cell and regulatory-T-cell axis reported in mouse skin repair makes wound healing a particularly relevant phase-specific endpoint (15). A narrow exposure margin, delayed immune recovery or impaired repair at effective exposures would weaken systemic-development claims. These findings could also constrain local strategies that reach protective skin compartments.

Atopic dermatitis needs an incremental-value test. A comparison of IRF4 perturbation with established IL-4Rα, IL-13, or JAK-pathway control is needed in endotype-defined human systems. The decisive question is whether IRF4 reduction changes an inflammatory output that is not adequately explained by upstream pathway blockade. Lack of added molecular or phenotypic benefit would support classification of IRF4 as a downstream marker or network component rather than an independent therapeutic target. Evidence of benefit would still require cell attribution, target engagement, and replication before a tractability claim could be made.

Testing selective modulation against whole-protein reduction is necessary before claiming superiority. Partner-, enhancer-, cell-, tissue-, dose-, and time-selective perturbations require the same efficacy and safety readouts, with recovery kinetics measured after withdrawal. Failure to preserve a predefined protective compartment would falsify the proposed selectivity advantage. Reporting species, tissue, cell state, perturbation type, target-engagement assay, and negative results would improve interpretability. These details would allow readers to distinguish lineage dependence from acute mediation and reversible modulation from developmental loss.

The evidence framework also needs prospective decision rules. Prespecification of the molecular change that constitutes IRF4 target engagement, the expected downstream response, the relevant phenotype, and the protective functions to be preserved would prevent *post hoc* reclassification. Results can then be classified prospectively. Target engagement without downstream change challenges the proposed IRF4 programme; downstream change without a disease-relevant phenotype questions functional importance; and phenotypic benefit without target engagement suggests an alternative mechanism. Using the same reagent and schedule for linked efficacy and safety measurements is preferable, because comparisons across unrelated genetic and pharmacological studies cannot establish a therapeutic margin. Public reporting of null findings, dose-response curves, tissue distribution, and recovery kinetics would reduce selective emphasis on positive phenotypes and permit independent assessment of whether a context boundary is reproducible.

Together, these experiments convert context dependence into prospective stop/go rules. A context is informative only when the responding cell, exposure, molecular programme, phenotype and recovery kinetics are measured together.

This review has methodological limits. The search was centred on PubMed and supplemented by PMC, citation chaining, and primary metadata records, with targeted updates through 21 July 2026. Authenticated exportable searches of Embase, Web of Science, and Scopus were unavailable. Relevance-ranked purposive screening was used, but complete prospective deduplication and exclusion counts were not recorded before targeted screening began. The review is therefore systematised and narrative rather than systematic, and no PRISMA flow diagram is presented. Statements about absent trials, delivery systems, or pharmacokinetic evidence mean that no study was identified in the accessible searches; they do not prove that no such study exists.

## 8 Conclusion and perspective

IRF4 broadens the inflammatory-mediator concept from secreted signals to intracellular regulators that specify the identity, magnitude, persistence, and resolution of inflammatory output. Its effects are encoded by abundance, partner choice, chromatin, metabolism, lineage, tissue, and disease phase. This architecture explains how the same factor can sustain pathogenic type 2 or type 17 programmes whilst supporting regulatory, humoral, antitumour, barrier, and repair functions elsewhere. Context dependence is therefore not a caveat to be acknowledged after the fact; it is the central mechanism that must be measured.

The causal-discrimination framework prevents expression and lineage requirement from being mistaken for therapeutic validation. Psoriasis is the most developed disease-linked preclinical IRF4 hypothesis identified in the accessible literature, yet the evidence remains concentrated largely in one study and does not establish human efficacy or reversal of established disease. AD remains a candidate epithelial and immune-network context. Conflicting results on maintenance of differentiated Th17 cells further show that acute dependence cannot be inferred from differentiation biology alone (35, 36). These evidence gaps, together with protective IRF4-dependent compartments, preclude a general claim that systemic inhibition will be effective or safe.

The decisive next step is independent perturbation in human tissues, using reversible IRF4 modulation at exposures that preserve predefined protective functions. Local and cell-, complex-, dose-, or time-selective strategies may create this separation, but each must demonstrate target engagement, programme selectivity, phenotypic benefit, and recovery after withdrawal. Translational success will depend less on suppressing IRF4 globally than on selectively reversing a pathogenic IRF4 programme without compromising immune or tissue homeostasis.

More broadly, IRF4 provides a test case for the thematic concept of novel mediators of inflammation and their regulation. An intracellular regulator should qualify as a mediator only when context-resolved perturbation links it to a defined molecular programme and disease-relevant phenotype. Therapeutic target status additionally demands reversible modulation, target engagement in the intended compartment, superiority or incremental value over existing pathway control, and an exposure-matched therapeutic window. This standard converts the language of context dependence into falsifiable development criteria.
